# Decreased Splenic CD4^+^ T-Lymphocytes in Apolipoprotein M Gene Deficient Mice

**DOI:** 10.1155/2015/293512

**Published:** 2015-10-12

**Authors:** Zhigang Wang, Guanghua Luo, Yuehua Feng, Lu Zheng, Hongyao Liu, Yun Liang, Zhonghua Liu, Peng Shao, Maria Berggren-Söderlund, Xiaoying Zhang, Ning Xu

**Affiliations:** ^1^Department of Cardiothoracic Surgery, Third Affiliated Hospital of Soochow University, Changzhou 213003, China; ^2^Comprehensive Laboratory, Third Affiliated Hospital of Soochow University, Changzhou 213003, China; ^3^Division of Clinical Chemistry and Pharmacology, Department of Laboratory Medicine, Lund University, 221 85 Lund, Sweden

## Abstract

Spleen T-lymphocytes, especially CD4^+^ T-cells, have been demonstrated to be involved in broad immunomodulation and host-defense activity in vivo. Apolipoprotein M gene (apoM) may have an important role in the regulation of immunoprocess and inflammation, which could be hypothesized to the apoM containing sphingosine-1-phosphate (S1P). In the present study we demonstrate that the splenic CD4^+^ T-lymphocytes were obviously decreased in the apoM gene deficient (apoM^−/−^) mice compared to the wild type (apoM^+/+^). Moreover, these mice were treated with lipopolysaccharide (LPS) and it was found that even more pronounced decreasing CD4^+^ T-lymphocytes occurred in the spleen compared to the apoM^+/+^ mice. The similar phenomena were found in the ratio of CD4^+^/CD8^+^ T-lymphocytes. After administration of LPS, the hepatic mRNA levels of tumor necrosis factor-*α* (TNF-*α*) and monocyte chemotactic protein-1 (MCP-1) were markedly increased; however, there were no statistical differences observed between apoM^+/+^ mice and apoM^−/−^ mice. The present study demonstrated that apoM might facilitate the maintenance of CD4^+^ T-lymphocytes or could modify the T-lymphocytes subgroups in murine spleen, which may further explore the importance of apoM in the regulation of the host immunomodulation, although the detailed mechanism needs continuing investigation.

## 1. Introduction

The apolipoprotein M gene (apoM), located on the major histocompatibility complex class III (MHC-III) region of chromosome 6p21.3, encodes a 26 Da glycoprotein of 188 amino acids [1]. Many genes in this region are related to the immune and inflammatory response [2, 3]. apoM is required for the formation of pre*β*-HDL particles and its presence in high-density lipoprotein (HDL) and pre*β*-HDL contributes to cellular cholesterol efflux in mice and humans [4]. It has been demonstrated that HDL could be a relevant player in both innate immunity and adaptive immunity. Such effects were explored mainly depending on the ability to finely modulate cholesterol bioavailability in immune cell [5, 6]. Moreover, the beneficial effects of HDL on the immune inflammation in vivo could also depend on the cargo of proteins and lipids carried by HDL which confer additional functions [7].

Recent investigations indicate that apoM is a natural carrier of sphingosine-1-phospate (S1P) in vivo, an important bioactive lipid mediator known to be associated with HDL [8]. S1P is a bioactive lysophospholipid with multiple effects on angiogenesis, lymphocyte trafficking, endothelial cell migration, and inflammation [9]. The activation of S1P receptor 1 (S1PR1) and the resulting signaling cascade could facilitate the egress of T-lymphocytes from lymphoid organs and modulate T-lymphocyte lineage [10, 11]. Moreover, S1PR1 is a switch factor that inhibits the development of anti-inflammatory regulatory T cells but drives differentiation of proinflammatory T helper type 1 cells (Th1) [12]. Previous investigations suggested apoM may also be involved in the inflammatory reaction and the potential immune-reactive properties [13–15].

The important role of apoM in modulating the inflammatory and immune response remains to be elucidated. With regard to immune function, the mammalian spleen plays important roles in the innate and adaptive immune systems. Analysis of the lymphocyte subgroups during development of inflammation could give important information on cell modifications occurring in the splenic lymphatic bed [16]. In addition, cytokines are immune regulators that were historically thought to be products solely of peripheral immune system. In the present study, we took advantage of wild type (apoM^+/+^) and apoM deficient (apoM^−/−^) mice to investigate whether the apoM affects the T-lymphocytes subgroups in the spleen with or without challenge of lipopolysaccharide (LPS). Additionally, we examined serum concentration and liver tissue mRNA levels of tumor necrosis factor-alpha (TNF-alpha) and monocyte chemotactic protein-1 (MCP-1).

## 2. Materials and Methods

### 2.1. Generation of apoM Gene Deficient Mice

**Figure 1:**
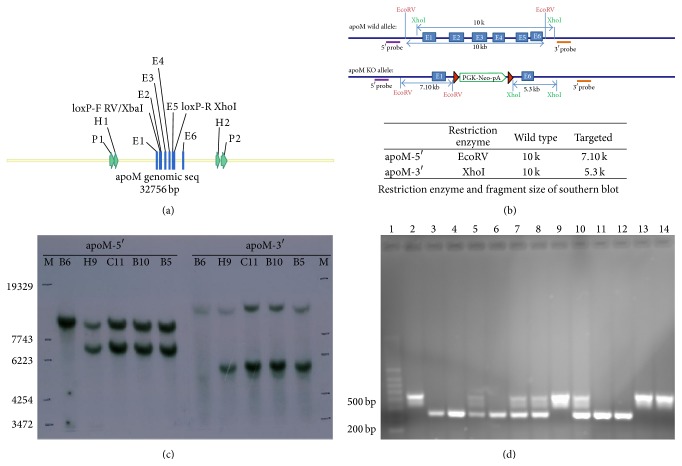
Generation of apoM deficient mice. (a) Replace apoM with a loxP-Neo-loxP cassette. (b) Southern blot analysis of 5′ and 3′ probes. (c) Results of Southern blot analyses of ES cells B5, H9, B10, and C11. B6 genomic DNA as negative control. (d) Genotyping generations of heterozygote/heterozygote and wild type crosses were performed by PCR of mouse tail DNA. Lanes 2, 9, 13, and 14 only showed bands at 558 bp, which was identified as apoM gene wild type; lanes 3, 4, 6, 11, and 12 only showed bands at 342 bp, which was identified as apoM gene deficient homozygotes; lanes 5, 7, 8, and 10 showed both 342 and 558 bp bands, which were identified as apoM gene heterozygotes.

The apoM^−/−^ mice were generated by homologous recombination with the help of Model Animal Research Center of Nanjing University (China). Briefly, BAC-retrieval methods were used to construct the targeting vectors. apoM gene was retrieved from a C57BL/6 BAC clone by using a retrieval vector that contained two homologous arms. After correct recombination, this vector contained 3.2 kb of the genomic sequence, including exon I–VI and 5 kb downstream sequences. Then, the entire exon II–V of apoM was deleted and replaced with a loxP-Neo-loxP cassette (Figure 1(a)). The neocassette was in the reverse-transcriptional orientation compared with the apoM gene. The targeting construct, which contained a neomycin-expression cassette for positive selection and a herpes simplex virus-thymidine kinase expression cassette for negative selection, was linearized with the NotI restriction enzyme and electroporated into C57BL/6N-derived B6/BLU ES cells. ES cell clones (192) were selected and verified for correct recombination using long-range PCR and Southern blot analysis (Figures 1(b) and 1(c)). Correctly targeted ES cells were injected into C57BL/6 blastocysts and transferred to pseudopregnant mice. Chimeric male mice were identified using PCR and were bred to C57BL/6 female mice to generate F1 offspring. The F1 offspring were crossed at least 2-3 generations of inheritance before the experiment. Subsequent genotyping generations of heterozygote/heterozygote and wild type crosses were performed by PCR of mouse tail DNA by using the common upstream primer 5′-GCCCAGACATGAAAACAGACCT-3′ and downstream primers 5′-CCACTCCCACTGTCCTTTCCTAAT-3′ and 5′-GGTACCATTCTAGCCCATAAGAATTAG-3′, which generated 558 and 342 bp bands from the apoM^+/+^ and apoM^−/−^ alleles, respectively (Figure 1(d)).

### 2.2. Animals and Experimental Procedure

Age- and body weight-matched male C57BL/6 apoM^+/+^ and apoM^−/−^ mice (23–25 g) at 6–8 weeks were used in the present study. The mice were housed in specific pathogen-free conditions and a temperature and humidity controlled environment (12 hrs light/dark cycle) with unlimited access to tap water and food. All the animal work was conducted in compliance with the recommendations on the guide for the care and use of laboratory animals and approved by the local ethical committee.

Mice were randomly assigned to receive intraperitoneal (i.p.) injection of the vehicle (pyrogen-free 0.9% saline; control group), 5 mg/kg LPS (LPS;* Escherichia coli* serotype O111:B4; Sigma-Aldrich, St. Louis, MO), or 10 mg/kg LPS for three times every 24 hrs. All mice were sacrificed at 96 hrs after the first i.p. injection. The blood samples and tissue samples were collected for further analysis. The spleens were prepared for the flow cytometry analyses immediately. The blood sample was centrifuged (12,000 ×g, 4°C, 20 min) and the serum was stored at −80°C until assay. All other tissues were snap-frozen in liquid nitrogen and stored at −80°C until processing.

### 2.3. Cell Preparation and Flow Cytometry Analysis

Spleens of the control group or LPS-challenged apoM^+/+^ and apoM^−/−^ mice were harvested aseptically and filtered through cell strainers to produce a single-cell suspension. After lysis of red blood cells, the spleen suspensions were washed three times with PBS and adjusted to a concentration of 1.0 × 10^7^/mL. To detect the changes in lymphocyte subgroups, the prepared splenocytes (1.0 × 10^7^/mL, 100 *μ*L) were incubated with 0.5 *μ*L each of fluorescently labeled antibodies for 30 minutes at 4°C protected from light and washed three times with PBS before analysis. Fluorescently labeled antibodies included FITC-conjugated anti-CD3, PE-Cy5 conjugated anti-CD4, and PE-Cy7 conjugated anti-CD8 (BioLegend, San Diego, CA). The stained cells were then analyzed by flow cytometry immediately (FC500, Beckman, USA).

### 2.4. Determinations of mRNA Levels of TNF-Alpha and MCP-1 in Livers

Total RNA was isolated from the liver tissue by the guanidinium thiocyanate method, and the quality of the samples was determined by the absorbance measurements at 260/280 nm. 2 *μ*g total RNA was reverse-transcribed to cDNA, using the first strand cDNA synthesis kit (Qiagen) according to the manufacturer's instructions. The mRNA levels were measured under real-time PCR using TaqMan assay for both target and reference genes. Mouse TNF-alpha and MCP-1 primer/probe sets were designed according to the information of GenBank, as presented in Table 1. GAPDH was used in separate tubes as reference control. Relative standard curves for all three templates were performed to compensate the efficiency of the PCRs. Quantification of TNF-alpha or MCP-1 mRNA levels was relative to the GAPDH mRNA levels. The real-time PCR reaction was performed on LightCycler 480 in a final volume of 25 *μ*L. Each well of a 96-well plate was containing 2.5 *μ*L 10 × PCR buffer, 1.5 *μ*L MgCl2 (25 mM), 0.5 *μ*L dNTPs (10 mM), 0.25 *μ*L Taq DNA polymerase, 0.1 *μ*L 100 *μ*M each primer and probe, 2 *μ*L cDNA, and ddH2O 17.95 *μ*L. Thermal cycling conditions for TNF-alpha and MCP-1 included the following steps: denaturation at 95°C for 3 min, followed by 40 cycles at 98°C for 5 s and 60°C for 27 s (for GAPDH, 60°C for 15 s).

### 2.5. Determinations of TNF-Alpha and MCP-1 in Serum

Based on the method of Luminex technology [17], the serum concentrations of TNF-alpha and MCP-1 were determined by using MILLIPLEX xMAP Mouse Cytokine/Chemokine Kit (Merck Millipore, USA). The procedure was performed according to the manufacturer's protocol. Quantification of these cytokines was performed using the Luminex 200 (Luminex Corp., USA) platform.

### 2.6. Statistics

Data are expressed as median with range. Statistical analyses were performed with the GraphPad Prism 6.0 software (GraphPad Software, Inc., San Diego, California, USA). Multiple comparisons were performed with one-way ANOVA, followed by Dunnett's multiple comparison test analysis. And comparisons between two groups were statistically evaluated by two-tailed Mann-Whitney *U* test. *p* values less than 0.05 were considered significant.

## 3. Results

### 3.1. The Splenic Total T-Lymphocytes (CD3^+^ Cells) and T-Lymphocyte Subgroups (CD4^+^ and CD8^+^ Cells) Were Determined by Flow Cytometry

**Figure 2:**
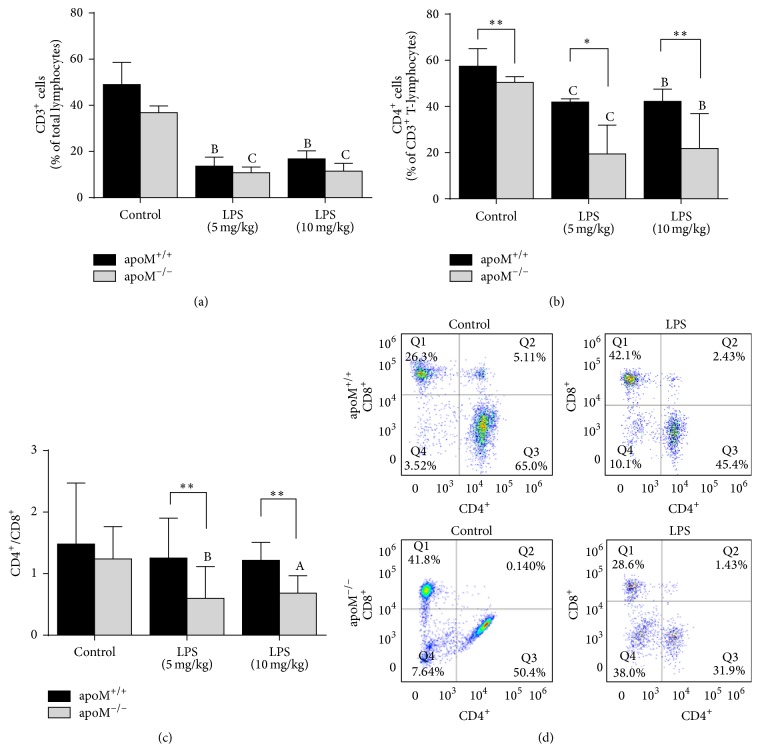
Distribution of splenic T-lymphocyte and its subpopulations in apoM^−/−^ and apoM^+/+^ mice with or without administration of LPS. (a) Total CD3^+^ T-lymphocytes were slightly lower in apoM^−/−^ than in apoM^+/+^ mice. (b) The proportion of CD4^+^ T-lymphocytes was obviously lower in apoM^−/−^ mice than that in apoM^+/+^ mice before LPS treatment. After administration of LPS, the CD4^+^ T-lymphocytes were dramatically decreased in the apoM^−/−^ mice, whereas CD4^+^ T-lymphocytes were only moderately decreased. (c) The ratio of CD4^+^/CD8^+^ was also obviously decreased in apoM^−/−^ mice compared to that in apoM^+/+^ mice after LPS treatment. (d) Representative FACS plots of splenic CD4^+^ and CD8^+^ T-lymphocytes in the apoM^+/+^ and apoM^−/−^ mice (*n* = 5~6, one-way ANOVA followed by Dunnett's test, ^A^ *p* < 0.05, ^B^ *p* < 0.01, and ^C^ *p* < 0.001 versus apoM^+/+^ or apoM^−/−^ control; Mann-Whitney *U* test, ^*∗*^ *p* < 0.05 and ^*∗∗*^ *p* < 0.01).

As shown in Figure 2(a), the total CD3^+^ T-lymphocytes were slightly lower in the apoM^−/−^ mice than those in apoM^+/+^ mice; after administration of LPS, the total CD3^+^ T-lymphocytes were dramatically decreased in both apoM^−/−^ mice and apoM^+/+^ mice (*p* < 0.001 and *p* < 0.01, resp.), although no statistical differences occurred in the apoM gene deficient mice compared to the wild type. Interestingly, the basal proportion of CD4^+^ T-lymphocytes in apoM^−/−^ mice was significantly lower than that in apoM^+/+^ mice (Figure 2(b), *p* < 0.01). After administration of LPS, at both concentrations of 5 mg/kg and 10 mg/kg, the proportion of CD4^+^ T-lymphocytes was significantly decreased in apoM^−/−^ and apoM^+/+^ mice (Figure 2(b), both *p* < 0.001). Furthermore, the decrease of CD4^+^ T-lymphocytes in apoM^−/−^ mice was much more than that in apoM^+/+^ mice (*p* < 0.05 and *p* < 0.01, resp.). There was no significant difference for the splenic CD8^+^ T-lymphocytes between apoM^+/+^ and apoM^−/−^ mice in the control group and LPS-challenged mice (data not show). As shown in Figure 2(c), the similar phenomena with CD4^+^ T-lymphocytes were found in the ratio of CD4^+^/CD8^+^ T-lymphocytes, although there was only slight decrease of basal CD4^+^/CD8^+^ ratio in apoM^−/−^ mice compared to apoM^+/+^ mice (*p* = 0.0556).

### 3.2. The Hepatic mRNA Levels and Serum Protein Levels of TNF-Alpha and MCP-1 in apoM^+/+^ and apoM^−/−^ Mice Were Determined by Real-Time PCR or Luminex Technology, Respectively

**Figure 3:**
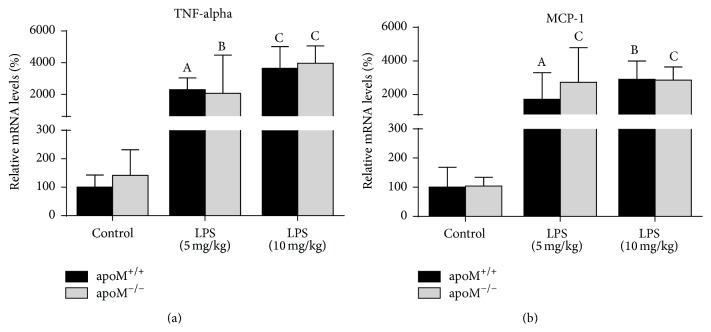
Hepatic mRNA levels of TNF-alpha and MCP-1 in apoM^−/−^ mice and apoM^+/+^ mice with or without LPS treatment. The mRNA levels of TNF-alpha (a) and MCP-1 (b) were similar in apoM^+/+^ mice and apoM^−/−^ mice before LPS administration. After LPS treatment the hepatic mRNA levels of TNF-alpha and MCP-1 were dramatically increased in both apoM^+/+^ and apoM^−/−^ mice. The mRNA levels in apoM^+/+^ mice in the control group were considered as 100% (*n* = 6~9, one-way ANOVA followed by Dunnett's test, ^A^ *p* < 0.05, ^B^ *p* < 0.01, and ^C^ *p* < 0.001 versus apoM^+/+^ or apoM^−/−^ control).

**Figure 4:**
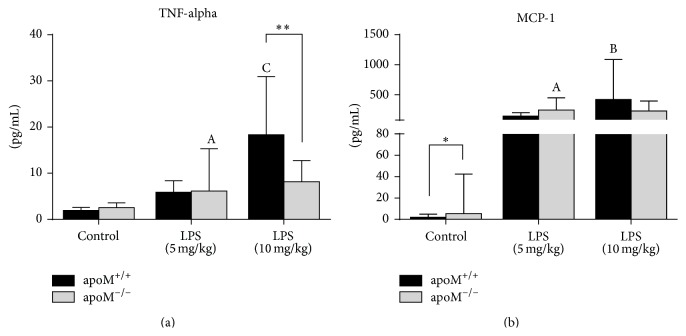
Serum protein levels of TNF-alpha and MCP-1 in apoM^−/−^ mice and apoM^+/+^ mice with or without LPS treatment. (a) The serum levels of TNF-alpha in both apoM^+/+^ and apoM^−/−^ mice were significantly increased after LPS treatment. (b) The basal MCP-1 levels were higher in apoM^−/−^ mice than in apoM^+/+^ mice. After LPS treatment, serum levels of MCP-1 were dramatically increased in both apoM^+/+^ and apoM^−/−^ mice, but there were no statistical differences between apoM^−/−^ mice and apoM^+/+^ mice (*n* = 6, one-way ANOVA followed by Dunnett's test, ^A^ *p* < 0.05, ^B^ *p* < 0.01, and ^C^ *p* < 0.001 versus apoM^+/+^ or apoM^−/−^ control; Mann-Whitney *U* test, ^*∗*^ *p* < 0.05 and ^*∗∗*^ *p* < 0.01).

As shown in Figure 3, the hepatic mRNA levels of TNF-alpha and MCP-1 were significantly increased in both apoM^+/+^ and apoM^−/−^ mice after administration of LPS, whereas there were no statistical differences between apoM^−/−^ mice and apoM^+/+^ mice. The serum levels of TNF-alpha in both apoM^+/+^ and apoM^−/−^ mice significantly increased after LPS administration (Figure 4(a), *p* < 0.001 and *p* < 0.05, resp.). However, the serum levels of TNF-alpha had no statistical difference between apoM^+/+^ and apoM^−/−^ mice in the control group and 5 mg/kg LPS-treated mice. The serum levels of TNF-alpha in apoM^−/−^ mice were extremely lower after 10 mg/kg LPS stimulation compared to apoM^+/+^ mice (*p* < 0.01). The serum levels of MCP-1 in both apoM^+/+^ and apoM^−/−^ mice were significantly increased after LPS administration (Figure 4(b), *p* < 0.01 and *p* < 0.05, resp.). The serum levels of MCP-1 in apoM^−/−^ mice were moderately higher than those in apoM^−/−^ mice (*p* < 0.05), while no statistical differences were observed between apoM^+/+^ and apoM^−/−^ mice after LPS treatment.

## 4. Discussion

CD4^+^ T-lymphocytes, also known as T helper cells, play an important role in orchestrating adaptive immune responses to various microbes. CD4^+^ T-lymphocytes recognize peptide-major histocompatibility complex (MHC) class II complexes presented by antigen-presenting cells (APCs) and differentiate into at least four lineages, Th1, Th2, Th17, and inducible T regulatory cells, which participate in different types of immune responses [18]. As a Toll-like receptor (TLR) 4 agonist, LPS stimulates TLR4 and then activates antigen-presenting cells through MyD88- and TRIF-dependent signaling pathways [19], which activates nuclear factor- (NF-) *κ*B, leading to release of inflammatory cytokines, and initiates CD4^+^ T-lymphocytes clonal expansion and functional differentiation [20].

apoM is the physiological carrier protein of S1P in HDL and apoM can deliver S1P to the S1PR1 [8], which can affect mammalian immunity through alterations of thymocyte emigration, differentiation of T-lymphocytes subgroups, and lymphocyte trafficking in lymphoid organs and other tissues [21]. It has been reported that HDL can promote the interaction of LPS with LPS-binding protein (LBP) favoring LPS clearance [22, 23], whereas the genetic knock-out apoM is associated with the absence of pre*β*-HDL particles and reduction of S1P in serum [4, 8].

In the present study, we demonstrated that the splenic CD4^+^ T-lymphocytes significantly decreased, whereas no obvious changes occurred on the CD8^+^ T-lymphocytes in apoM^−/−^ mice compared to apoM^+/+^ mice. After administration of LPS, total CD3^+^ T-lymphocytes were significantly decreased in both apoM^−/−^ and apoM^+/+^ mice, although there were no statistical differences between apoM^−/−^ and apoM^+/+^ mice. However, the splenic proportion of CD4^+^ T-lymphocytes was dramatically decreased in apoM^−/−^ mice and there was only moderate decrease of CD4^+^ T-lymphocytes in apoM^+/+^ mice (Figure 2(b)). The similar phenomenon was found for the ratio of CD4^+^/CD8^+^ in apoM^−/−^ mice (Figure 2(c)). These findings coupled with systemic inflammation and sepsis challenged by LPS caused marked lymphopenia that resulted in the decrease of total splenic T-lymphocytes and CD4^+^/CD8^+^ T-lymphocytes ratio [24]. It has been previously reported that apoA-I, a major protein contained in HDL, is essential for HDL formation and function. With lipid-free apoA-I injection, male LDLr^−/−^ and apoA-I^−/−^ DKO mice exhibited reduced infiltrates and inflammation in skin and lymph nodes compared with LDLr^−/−^ SKO mice, including an increase in regulatory T cell expansion within the CD4^+^ T-lymphocytes pool [25]. The genetic approaches to alter the function of S1PR1 have established that S1PR1 is the main S1P receptor that regulated T-lymphocytes trafficking: T-lymphocytes from S1PR1-deficient mice failed to egress from the thymus and peripheral lymphoid organs, whereas S1PR1-transgenic T-lymphocytes preferentially distributed to the blood rather than lymphoid organs [26]. apoM-S1P is able to activate the S1PR1 and affect the function of endothelial cells [8]. Meanwhile apoM^−/−^ mice with the congenital low S1P level impaired the ability of activating S1PR1 in the endothelial cells, which may reduce the trafficking of T-lymphocyte, especially CD4^+^ T-lymphocytes, between the thymus and the spleen, and result in the decrease of splenic CD4^+^ T-lymphocytes or the CD4^+^ T-lymphocytes would distribute to the blood rather than the spleen. A recent paper published in Nature [27] demonstrated that the absolute counting of CD4^+^ and CD8^+^ T-lymphocytes and CD19^+^ B-lymphocytes in peripheral blood and lymph was apoM^−/−^ mice, and the quantification of splenic CD4^+^ and CD8^+^ cells was similar in apoM^+/+^ and apoM^−/−^ mice without LPS administration. Meanwhile, in our study, we observed that the percentage of splenic CD4^+^ T-lymphocytes in the wild type was higher than that in apoM^−/−^ mice before LPS administration. Then, we studied the percentage of CD3^+^ of total lymphocytes and CD4^+^ of total CD3^+^ T-lymphocytes in the spleen after LPS administration. We showed that the percentage of CD3^+^ and CD4^+^ T-lymphocytes was decreased in the spleen after the treatment of LPS. Moreover, we found that even more pronounced decreasing splenic CD4^+^ T-lymphocytes occurred in apoM^−/−^ mice compared to apoM^+/+^ mice. Timothy Hla and his colleagues used different strategy of apoM gene knockout model from us [8, 28], which was designed to delete 39 bp of the endogenous apoM sequences in exon 2 and insert a neomycin resistance-encoding cassette in the apoM locus. Different apoM gene knockout strategies and experimental design may attribute to these contradictive results in the paper. apoM^+^HDL inhibited the bone marrow proliferation of lymphocyte progenitors, through the activation of S1PR1 on these cells, while the increase of circulating lymphocytes was found in apoM^−/−^ mice with the exaggerated autoimmune neuroinflammatory response, whereas overexpression of apoM was protective [27]. We speculate that apoM could restrain the bone marrow proliferation of lymphocyte progenitors and the cell cycle of T-lymphocytes from the secondary lymphoid organs to the periphery blood even after the administration of LPS, so the wild type harbored more CD4^+^ T-lymphocytes in the murine spleen than apoM^−/−^ mice. Taking together, our present study may provide certain evidence that apoM might facilitate the maintenance of splenic CD4^+^ T-lymphocytes. Moreover, apoM would modify the T-lymphocytes subgroups in the murine spleen, whereas the splenic CD8^+^ T-lymphocytes would remain unchanged between apoM^+/+^ and apoM^−/−^ in these conditions. Thus, it would be of great interest to determine the differential effects of apoM on various CD4^+^ T-lymphocytes and their subpopulations to gain insights into their roles in different tissue microenvironment.

TNF-alpha, as a member of a pleiotropic inflammatory cytokine, responds to microbes, especially the LPS, and stimulates the acute phase reaction. It is produced chiefly by activated macrophages [29], although it can be produced by many other cell types such as CD4^+^ lymphocytes, neutrophils, mast cells, and eosinophils [30]. Monocyte chemotactic protein-1 (MCP-1), also referred to as the chemokine (C-C motif) ligand 2 (CCL2), is a small cytokine that belongs to the CC chemokine family. MCP-1 recruits monocytes, memory T cells, and dendritic cells to the sites of inflammation [18, 31]. In the present study, as expected, after LPS treatment, the hepatic mRNA levels and serum protein levels of TNF-alpha and MCP-1 were dramatically increased in both apoM^−/−^ mice and apoM^+/+^ mice; however, there were no extremely differences found between apoM^−/−^ mice and apoM^+/+^ mice. In continuing experiments we will try to investigate other cytokines in the apoM gene deficient mice with or without LPS treatment. It has been reported that high serum levels of endotoxins were found in the patients suffering from common variable immunodeficiency (CVID), which suggest that CD4^+^ T-lymphocytes dysfunction was restricted to bacteria-specific and not virus-specific CD4^+^ T-lymphocytes, including the reduced capacity to proliferate and to produce IFN-*γ* and IL-2 [32]. Persistent inflammation, impaired CD4^+^ T-lymphocytes activation, and caused T-lymphocytes exhaustion may be associated with decreased survival in elderly patients and mice after sepsis [33]. Current investigation suggests that T-lymphocytes exhaustion is a newly recognized pathophysiologic mechanism of immunosuppression in sepsis that results in failure to activate macrophages and eradicate invading pathogens, thereby increasing susceptibility to secondary infections, leading to prolonged inflammation [34]. The significant reduction of TNF-alpha after higher dosage of LPS treatment in apoM^−/−^ mice might be a partial consequence of the depletion of CD4^+^ T-lymphocytes in the spleen and the exacerbation of inflammatory condition. This finding is in agreement with the reduction of proliferative capacity of CD4^+^ memory cells suppressed by persistent antigen presentation extending beyond the priming phase [35].

It is concluded that the present study shows the decrease of splenic CD4^+^ T-lymphocytes and low ratio of CD4/CD8 in apoM gene deficient mice compared to the wild type, which suggests that apoM might facilitate the maintenance of CD4^+^ T-lymphocytes or modify the T-lymphocytes subgroups; it can even accumulate the splenic CD4^+^ T-lymphocytes after LPS administration in the murine spleen. These results may further explore the importance of apoM on the regulation of immune responses, although the detailed mechanism needs continuing investigation.

## Figures and Tables

**Table 1:** Sequences of primers and probes.

Gene	Primer/probe	Sequence (5′-3′)
TNF-alpha	Froward primer	tgacaagcctgtagcccacg
	Reverse primer	ttgtctttgagatccatgccg
	Probe	FAM-cgtagcaaaccaccaagtggaggagc-TAMRA

MCP-1	Froward primer	gctggagagctacaagaggatcac
	Reverse primer	ccttcttggggtcagcacag
	Probe	FAM-cagcaggtgtcccaaagaagctgtagtt-TAMRA

GAPDH	Froward primer	tcttgtgcagtgccagcct
	Reverse primer	tgaggtcaatgaaggggtcg
	Probe	FAM-aggtcggtgtgaacggatttggc-TAMRA
